# Intracranial pressure-flow relationships in traumatic brain injury patients expose gaps in the tenets of models and pressure-oriented management

**DOI:** 10.3389/fphys.2024.1381127

**Published:** 2024-08-12

**Authors:** J. N. Stroh, Brandon Foreman, Tellen D. Bennett, Jennifer K. Briggs, Soojin Park, David J. Albers

**Affiliations:** ^1^ Department of Biomedical Informatics, University of Colorado Anschutz Medical Campus, Aurora, CO, United States; ^2^ Department of Bioengineering, University of Colorado Denver |Anschutz Medical Campus, Denver, CO, United States; ^3^ Department of Neurology and Rehabilitation Medicine, University of Cincinnati, Cincinnati, OH, United States; ^4^ Gardner Neuroscience Institute, University of Cincinnati, Cincinnati, OH, United States; ^5^ Pediatric Intensive Care, Children’s Hospital of Colorado, Aurora, CO, United States; ^6^ Department of Biomedical Informatics, Columbia University, New York, NY, United States; ^7^ Department of Neurology, New York Presbyterian/Columbia University Irving Medical Center, New York, NY, United States

**Keywords:** Intracranial hemodynamics, traumatic brain injury, neurocritical care, Hagen-Poiseuille flow, cerebral autoregulation

## Abstract

**Background:** The protocols and therapeutic guidance established for treating traumatic brain injury (TBI) in neurointensive care focus on managing cerebral blood flow (CBF) and brain tissue oxygenation based on pressure signals. The decision support process relies on assumed relationships between cerebral perfusion pressure (CPP) and blood flow, pressure-flow relationships (PFRs), and shares this framework of assumptions with mathematical intracranial hemodynamics models. These foundational assumptions are difficult to verify, and their violation can impact clinical decision-making and model validity.

**Methods:** A hypothesis- and model-driven method for verifying and understanding the foundational intracranial hemodynamic PFRs is developed and applied to a novel multi-modality monitoring dataset.

**Results:** Model analysis of joint observations of CPP and CBF validates the standard PFR when autoregulatory processes are impaired as well as unmodelable cases dominated by autoregulation. However, it also identifies a dynamical regime -or behavior pattern-where the PFR assumptions are wrong in a precise, data-inferable way due to negative CPP-CBF coordination over long timescales. This regime is of both clinical and research interest: its dynamics are modelable under modified assumptions while its causal direction and mechanistic pathway remain unclear.

**Conclusion:** Motivated by the understanding of mathematical physiology, the validity of the standard PFR can be assessed *a*) directly by analyzing pressure reactivity and mean flow indices (PRx and Mx) or *b*) indirectly through the relationship between CBF and other clinical observables. This approach could potentially help to personalize TBI care by considering intracranial pressure and CPP in relation to other data, particularly CBF. The analysis suggests a threshold using clinical indices of autoregulation jointly generalizes independently set indicators to assess CA functionality. These results support the use of increasingly data-rich environments to develop more robust hybrid physiological-machine learning models.

## 1 Introduction

Clinical management is essential for improving patient neurological outcome following traumatic brain injury (TBI), an contributor to tens of thousands of fatalities in the United States annually (https://wonder.cdc.gov/mcd.html). TBI patients risk insults such as elevated intracranial pressure (ICP) and cerebral ischemia, compounding the prospect of secondary injuries such as hemorrhage and hypoxic tissue death. Potential contributors to such problems include impaired or overburdened cerebral autoregulation (CA), a collection of endogenous perfusion control mechanisms that modify cerebral vessel diameter (Claassen et al., 2021). Observable pressures–ABP and ICP–play a critical role in guiding TBI intervention strategies (Stocchetti and Maas, 2014). Meanwhile, blood transport, as quantified by cerebral perfusion and cerebral blood flow (CBF), is not typically measured and does not currently factor in care protocols. This work focuses on a core tenet of TBI management (Bouzat et al., 2013): the relationship between commonly observed hemodynamic pressures and the associated, but typically unobserved, perfusion. Current TBI protocols set thresholds for ICP or cerebral perfusion pressure (CPP, the mean ABP-ICP difference) to improve categorical clinical outcomes (Carney et al., 2017; Kochanek et al., 2019). The approach is contentious (e.g., Asgeirsson et al., 1994; Helbok et al., 2018) and lacks personalization (Stroh et al., 2021a) despite improvements afforded by integrating e.g., brain tissue oxygenation (P_bt_O_2_) (Chesnut et al., 2020), demographics, or disease severity (Sorrentino et al., 2012). Protocol objectives aim to maintain adequate perfusion for metabolic processes while minimizing the risk of secondary insults such as hyperemia and vascular barotrauma (Haddad and Arabi, 2012; Kirkman and Smith, 2014). Recent work (Pelah et al., 2023) identified dissociation between optimal CPP and optimal flow and oxygenation, further highlighting the insufficiency of pressure-targeted approaches. Directly or indirectly, pressure-guided protocols address perfusion, broadly referred to here as cerebral blood flow. Nevertheless, perfusion and CBF are infrequently observed despite both invasive (Vajkoczy et al., 2000; Rosenthal et al., 2011) and noninvasive (Schmidt et al., 2001; White and Venkatesh, 2006) observation methods.

CBF and observable pressures interact with the pressure-responsive vasocontrol and flow-regulatory mechanisms (Claassen et al., 2021) of CA that influence the pressure-flow relationship (PFR). The role of CA is implicit in the pressure reactivity index (PRx) (Czosnyka et al., 1997; Czosnyka et al., 2017), a quantification of CA function from ABP and ICP. Explicitly, the mean flow index (Mx) (Czosnyka et al., 1996) gauges CA via correlation of middle cerebral artery blood velocity (or its flow (Blanco and Abdo-Cuza, 2018)) with CPP (or ABP, assuming ICP is constant). Existing studies associate favorable patient outcome with Mx or PRx lying below 0.3, establishing a heuristic indicator of CA functionality (Lang et al., 2002; Sorrentino et al., 2012; Riemann et al., 2020).

**FIGURE 1:**
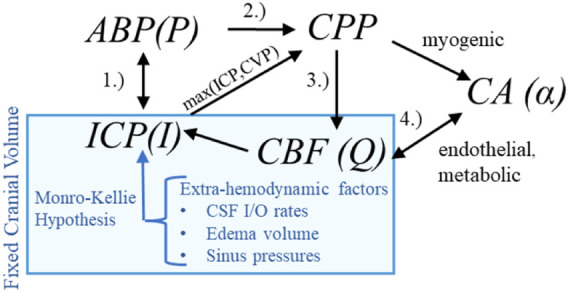
Hemodynamics relationships associated with ICP In the first relationship (1.), ABP acts as the system inflow pressure with ICP opposing outflow pressure when it exceeds central venous pressure (CVP). In the second (2.), the ABP-ICP difference defines the background pressure gradient (CPP). The third (3.) is the pressure-flow relationship: CBF is determined by the pressure gradient subject under vasoregulation and other influences. The relationship (4.) identifies the co-dependence of functioning CA and CBF (or available oxygen/nutrients beyond the scope of this discussion).

Hemodynamic pressures (ABP, ICP), perfusion (CBF), and assemblage of CA processes form an inter-dependent system of intracranial hemodynamics (ICHD, Figure 1). In this system, Mx and PRx quantify specific aspects of ICHD interaction. A more complete description of ICHD includes cranial volume capacity, cranial blood volume, and additional extra-hemodynamic factors. Cerebrospinal fluid (CSF) and sagittal sinus pressures are important constituents of intracranial volume (Davson et al., 1970; Kosteljanetz, 1986; Czosnyka et al., 2004; Czosnyka et al., 2012) within the classical Monro-Kellie framework (Wilson, 2016). This investigation omits the dynamical contributions of these extra-hemodynamic components, which are neither considered in ICP/ICP-guided protocols nor observable without non-standard patient manipulations. Conceptually, this ICHD model (discussed below) asserts that perfusion is governed by CPP dynamics mitigated by CA processes.

### 1.1 Pressure-flow relationships

Defining both simulation models (e.g., (Kashif et al., 2012)) and clinical indices as PRx, Mx, and CPPopt (Aries et al., 2012; Tas et al., 2021) requires assuming relationships among pressure, flow, and autoregulation. In elementary fluid settings, for example, increases in pressure difference imply increases in flow co-determined by changes in vessel diameter. The fluid relationship synthesized with knowledge of CA’s mitigating function motivates PFR hypotheses according to sign and potential causal direction:

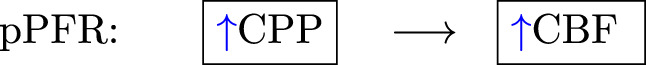

(1)



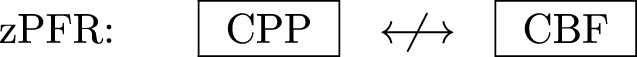

(2)



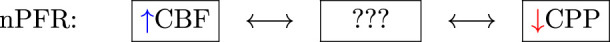

(3)



The positive PFR (pPFR, Eq 1) posits that CBF results from pressure-driven processes regulated by variable vasoresistance. This pressure-passive situation is associated with autoregulatory impairment and serves as a baseline hypothesis in this work. The pPFR, for example, is assumed by cerebrovascular resistance (CVR, the mean ratio of CBF to CPP, i.e., CBF = CPP/CVR (Nordström et al., 2013; Powers, 2016; Canac et al., 2020)) as a relative measure of CA function. This work operates within the common perspective that CA modulates CVR in response to various factors, including non-hemodynamic and volumetric influences omitted in the model. Under fixed CPP, flow increase is mathematically associated with decreased CVR and tied to CA-driven vasodilation. However, CVR is expected to remain positive so that CBF changes are positively or neutrally related CPP changes. Increased CBF under decreased CPP requires the vasodilation to be modeled by negative resistance.

The zero PFR (zPFR, Eq. 2) indicates decoupled CBF and CPP, presumably by functional autoregulation yielding no net correlation between flow and pressure. The negative PFR (nPFR, Eq. 3) is an alternate hypothesis comprising anti-correlated pressure-flow dynamics resulting from unspecified processes. The dynamics of ICHD under zPFR and nPFR violate the positive pressure-flow association enforced by pPFR-based models, and these cases are therefore expected to be poorly modeled.

This study hypothesizes that the functional CA of zPFR represents equilibrium between positive and negative PFRs over 2-h timescales with neither being dominant. Namely, impaired CA corresponds to pPFR and functional CA to zPFR, while the nPFR is not expected to persist over such long timescales.

### 1.2 Purpose and outline

This work examines the validity of assumptions made by conceptual hemodynamics models which relate observable pressures to the perfusion quantities affected by pressure-guided TBI management protocols. The lack of necessary ICHD data has prevented the validation at multi-hour timescales of the pressure-flow relationships that underlie pressure-oriented TBI therapies and autoregulatory measures. This work exploits a single-center multi-modal monitoring (MMM) observation of neurocritical patients to investigate model assumption consistency with observed signals in clinically relevant TBI cases. It assesses model sufficiency in application to clinically relevant cases. Its goals are to compute assumptions’ failure, to bring knowledge limits into focus, and to propose practicable model domain improvements.

The analysis of this work uses model-aided PFR categorization of clinical data to identify patterns of intracranial hemodynamics inaccurately represented by pPFR assumptions. Cases with persistent negative pressure-flow association (nPFR) compose a sizable portion of examined data; alternate assumptions not presently formulated are required to inform neurocritical care of patients presenting these dynamics. Empirical characteristics of CA indexes associated with pPFR and non-pPFR data indicate that pressure-flow relationships generalize qualitative assessment of autoregulatory function and suggests a pathway for including CBF data into the decision support process.

## 2 Methods

This research explores PFRs by analyzing neurocritical patient data through model-simulated hemodynamics. These vital aspects are presented prior to defining the experiments and metrics used to assess PFRs. Simulations generate simulated observations whose appropriateness categorizes pressure-flow association of the data. This hypothesis-driven assumes that PFR timescales manifest between timescales of 1–2 min and 2 h.

### 2.1 The neurocritical care dataset

The University of Cincinnati obtained continuous multimodality monitoring data from neurointensive TBI patients between 2014 and 2019 (Table 1). The collection occurred with prior consent under local institutional review board authorization (UCIRB #18–0743), These single-center data include concurrent records of ABP and ICP (125 Hz), plus brain tissue perfusion (1 Hz), brain tissue oxygenation (P_bt_O_2_, 1 Hz), and intracranial temperature (ICT, 0.5–2 Hz) observed by a Bowman probe (https://hemedex.com/products/bowman-perfusion-monitoring- system/). Further patient data include extra-ventricular drainage (EVD) transducer observations (which may be open or clamped), systemic monitors (EtCO_2_, central venous pressure, temperature), temperature management system use, and PRx computed bedside through Moberg CNS monitor systems (https://www.moberg.com/products/cns-monitor). The recording and storage process discretize patient data into epochs of irregular length that were analyzed separately. EVD observation of ICP was not used because records lack clamp status and calibration timeseries needed for hour-scale analysis.

**TABLE 1:** Cohort description of University of Cincinnati MMM dataset Data described as mean  ±  standard deviation, median [quartile range], or proportion (%) as appropriate.

Variable	Value (N = 25)
Demographics and Injury Characteristics	
Age (years)	40.2 ± 17.7
Sex (male)	20 (80%)
Injury mechanism	
Motor Vehicle Collision	12 (48%)
Fall	10 (40%)
Other	3 (12%)
Δt Injury to Admission (hrs)	1.5 [0.8, 2.0]
Admission Status	
Glasgow Coma Score (GCS)	3 [3, 5]
Motor Subscore	1 [1, 3]
Unreactive Pupil (1 or both)	11 (44%)
Pre-hospital Hypoxia	4 (16%)
Pre-hospital Hypotension	0 (0%)
Rotterdam CT Score	4 [3, 5]
Injury Severity Score	25.7 ± 9.5
Encounter Therapies	
Mechanical Ventilation	25 (100%)
Decompressive Hemi-crainectomy	9 (36%)
Monitoring Data	
Δt Injury to Moberg CNS data (hrs)	13.1 [7.2, 17.7]
Duration of Moberg CNS data (hrs)	32.1 [22.5, 57.3]
ICP Monitoring	16 (64%)
Δt Injury to ICP Monitoring (hrs)	6.6 [5.5, 14.6]
ICP Monitor Type	
Parenchymal Monitor	11 (69% of 16)
External Ventricular Drainage Catheter	0 (0%)
Both	4 (25% of 16)
ICP Available Waveform Signal Data	16 (100% of 16)
ICP Available Numeric Data	16 (100% of 16)
P_bt_O_2_ Monitoring	16 (64%)
P_bt_O_2_ Available Numeric Data	16 (100% of 16)
Outcome	
Hospital Length of Stay (days)	12.8 [11.8, 20.1]
In-Hospital Mortality	8 (32%)
Withdrawal of Life-Sustaining Therapy	6 (75% of 8)
6-Month Mortality	10 (40%)
6-Month Glasgow Outcome Scale-Extended	3 [1, 6]

The dataset contains synchronized bedside monitoring system records and a time record of categorical interventions (e.g., suctioning, posture change, or sedation change). This work investigates dynamics *among* available monitored signals without access to electronic health records (EHRs) of patient injuries, medication dosages, intervention details, or patient laboratory data. The single-site cohort received standardized care, thereby minimizing variances of therapeutic influence on ICHD through sedation, pressor use, fluid management, and ventilator settings (such as positive end-expiratory pressure or PEEP) (Slupe and Kirsch, 2018). Endnotes of this work address data access.

### 2.2 Simulating hemodynamics and metrics

This study outlines a common conceptual ICHD framework used across various models to ensure generalizability. The system comprises ABP (P) , ICP (I) , CA processes (α) , and CBF (Q) whose interactions appear in Figure 1. The model hypothesizes that blood flow results from the arteriovenous pressure gradient and interactive CA processes. More complex hypotheses considering cerebrospinal fluid (Czosnyka et al., 1997) or volume distributions (Ryu et al., 2015) are excluded; their associated models are difficult to identify and are too computationally expensive for hour-scale simulation (Stroh et al., 2021b).

The pressure gradient (∇p) is the assumed driving force of the system, and its average is the analogue of CPP. Ignoring spatial heterogeneity of vasculature, the zero-dimensional compartmental ICHD system reduces to:

$$Q=\alpha_{1}\nabla p+\alpha_{2}\frac{d}{dt}\left(\nabla p\right)=F\left(\nabla p,\alpha \right)$$ (4)

where components of the vector $\alpha =\left(\alpha_{1},\alpha_{2}\right)$ parametrize CA effects on vessel bed conductance and compliance, respectively. Components of α are assumed to be non-negative due to assumption of pPFR (Eq. 1) except as transient behavior. The compartmental model Eq. 4 is adopted in various models (Kashif et al., 2012; Fanelli et al., 2019; Imaduddin et al., 2019) or approximates those: with more complex CA (Ursino and Lodi, 1997; Ursino and Lodi, 1998), with CSF and sinus volume dynamics (Czosnyka et al., 1997), with spatially-distributed volume components (Hu et al., 2007; Ryu et al., 2015; Wang et al., 2019), or with venous dynamics (Spronck et al., 2012).

Temporal averaging produces the common CVR definition $\alpha_{1}=\nabla p/Q$ because the compliant storage term of Eq. 4 is negligible in relation to the total flow. The time-averaging in differential form relates changes in CPP to changes in CBF, $\frac{d}{dt}Q=\alpha_{1}\frac{d}{dt}\nabla p$ , which is relevant to analysis through correlation. Importantly, it applies to nPFR dynamics with the inclusion of a negative sign while maintaining α>0 . *The relationship between this sign and PFR choice is exploited to experimentally assesses model assumptions (pPFR).*

This work uses Eq. 4 to simulate 1-min average ICP from pulsatile ABP and CBF signals under constant CA processes, as in (Kashif et al., 2012; Fanelli et al., 2019). This model has been validated using shorter non-invasive CBF velocity measurements from the middle cerebral artery. Longer, non-pulsatile perfusion timeseries available to the present study require use via model inference (detailed in Supplementary Section S1). The technique estimates CBF from CPP by optimizing non-negative control parameters α , ensuring consistency between Eq. 4 and CPP data at each step.

#### 2.2.1 Posterior correction and hypothesis assessment

Model-estimated CBF is recalibrated to account for uncertainty of model-to-observation CBF correspondence and to test the asserted PFR hypothesis. Global CBF estimates (in mL/s) must be compared with local perfusion data (in mL/hg/min) by overcoming personalized biases and scale differences arising from patient-specific anatomy, injury, and probe location. Scale and bias are adjusted by fitting CBF estimates to perfusion data with a linear correction m⋅CBF+b . Note that m is identified by minimizing the root mean squared error (RMSE) between CBF and perfusion scaled by their respective variances. The resulting values of m are therefore normalized to the observed data. This allows for a uniform comparison across experiments where perfusion ranges vary in magnitude due to probe location heterogeneity.

The *sign* of the correction slope m gauges whether model-generated CBF trajectories and observed perfusion have the same orientation, thereby identifying the fitness of the pPFR assumption for the observed pressure-flow dynamics. The slopes provide a compact, qualitative assessment of experiment PFRs and their associated dynamics. Positive slopes (m>0) signify CA impairment with perfusion data aligned with pressure-passive CBF assumptions (pPFR). Negative slopes (m<0) signify opposition of perfusion data to these assumptions arising from nPFR-driven dynamics. Near-zero slopes identify no clear net CPP-CBF relationship over the 2-h experiment. PFRs, or dynamical regimes, are categorized by the slope m of the correction for each experiment: pPFR cases are identified by m>0.2 ; nPFR by m<0.2 ; and zPFR by |m|≤0.2 . Parameter thresholding near |m|=0.2 conservatively defines the zPFR identity to approximate a balance between specificity of zPFR and sensitivity to nPFR and zPFR identities (see Supplementary Section S1).

#### 2.2.2 Mean flow index calculation

Mean flow (Mx) index quantifies CA by gauging the relation of CBF changes to those CPP. The averaging and correlation windows affect the timescale and resolution of Mx, and there is no consensus choice for these parameters (Olsen et al., 2022). This work calculates Mx using 6-min windows defined by 30-sample correlations of 12-s averages of CBF and CPP data. The choice stabilizes the index (Mahdi et al., 2017) while incorporating sub-minute pressure-flow variations inaccessible to the minute-scale model.

### 2.3 Experiment selection

Experiments in this work aim to characterize PFRs by analyzing numerical simulations, clinical indexes (Mx and PRx), patient observations during intervals satisfying data requirements. From the available patient data, fourteen patients were identified with the joint ABP, ICP, and perfusion recordings needed to perform data experiments; Supplementary Section S2) summarizes these patient epochs. Intervals of 100–140 min (nominally, 2 h) were algorithmically extracted from these patient records by excluding periods of missing data while ignoring gaps in perfusion data up to 10 min that may result from probe calibration. The extraction identified 193 patient-data intervals, which were further screened to omit those with perfusion sensor data flagged as faulty or where signals violated quality control thresholds. This filter removed intervals where: i) ABP is negative or identified as an outlier among the 99.8^th^ percentile of the data; ii) perfusion is negative, exceeds 130 mL/hg/min globally, or has a 5-min mean over 95 mL/hg/min; or iii) ICP is negative anywhere or continuously exceeds 100 mm Hg for longer than 5 min. The set remaining defines 83 2-h intervals from 11 patients; these are used for computational experiments (Supplementary Section S3). The 2-h duration maximizes the likelihood of a posture change or suctioning occurring within each experiment; such events occur in the data with a median frequency of about 1.5 h. These disturbance events prompt hemodynamic responses that facilitate PFR identification.

## 3 Results

This work extracts, checks, and contextualizes assumed ICHD relationships to gain understanding that may improve care of TBI patients. It identifies PFRs for individual patient-intervals, providing additional information about patient hemodynamics and autoregulation status. Section 3.1 examines hemodynamics data and clinical indices with an emphasis on pressure flow relationships over timescales of days. Section 3.2 categorizes PFRs of the data over 2-h patient-interval experiments using model simulated CBF. Section 3.3 assesses relationships between dynamics, indices, and model estimation for the numerical experiment intervals. The closing results section briefly synthesizes results into main conclusions.

### 3.1 Data-oriented analysis of dynamics via indices

Tabulated statistics of PRx and Mx over day-scale data (Supplementary Table S2) suggest differences in PFR regimes as measured by the CA functionality indices. The Mx mean values are negative in only 6 patient-epochs, while the median value is ∼ 0.36. This median Mx exceeds the threshold of Mx = 0.3 commonly identifying impaired CA (Czosnyka et al., 1996; Olsen et al., 2022) and suggests a statistical dominance of a pressure-driven flow (pPFR) among these records. However, over one-quarter of the patient-epochs show decoupled perfusion and CPP (|Mx|<0.15) when summarized over tens of hours. This suggests that CA is functional in a sizable portion of cases, although variability is large. On shorter timescales of several hours, locally summarized index values may be more extreme: variability exists both within and across patients. The PRx values associated with neutral Mx values $\left(\bar{\text{PRx}}=0.08\right)$ are significantly smaller (one-sided p=0.007 ) than the remainder $\left(\bar{\text{PRx}}=0.28\right)$ . These neutral values indicate active CA influences when most data are examined over timespans of hours or less.

### 3.2 Model-estimated CBF

Eighty-three experiments simulate CBF from ABP and ICP data using a model to enforce positive pressure-flow association (pPFR) under CA stationary at 1-min timescales. The aptness of pPFR for each 2-h experiment is identified from the posterior calibration of estimated CBF to observed perfusion. Linear corrections that preserve the model trajectory orientation identify pPFR while those that change it indicate a negated CPP-to-CBF relation and therefore identify nPFR. Optimal adjustments that either approximately nullify orientation or fail to calibrate the model to data identify zPFR. Among the 83 experiments representing 11 patients, the distribution of PFRs categories is as follows: 46% were identified with pPFR, 28% with zPFR, 27% with nPFR. Supplementary Section S3 chronicles experiment results and the associated summary (Supplementary Section S4) shows these proportions to be independent of probe quality diagnostics.

#### 3.2.1 Experiments identified with positive PFR

**FIGURE 2:**
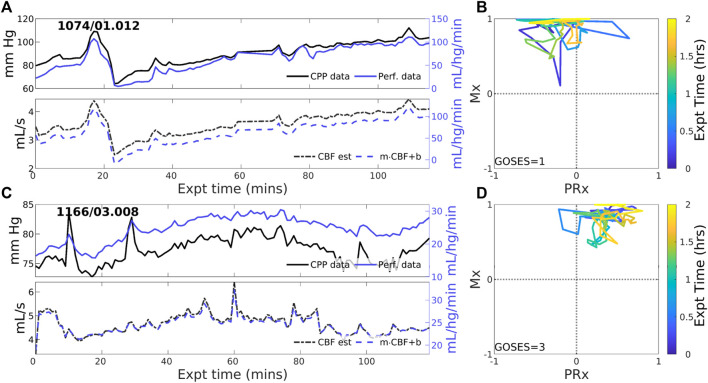
Two example experiments with positive PFR. (upper panels of **(A,C)**) Experiments in this set show consistent, positive coordination between CPP (black) and perfusion (blue) observational data. (lower panels) Model estimated CBF (black, dashed) follows changes in CPP; posterior correction (blue, dashed) preserves the original orientation of the estimate without a change in sign. (panels **(B,D)**) The associated trajectories in correlation indices show strongly positive Mx values, while PRx may be variable including sign changes.

Overall positive coordination between CBF and CPP (pPFR) is dominant in 38 of 83 experiments (46%). Cases identified by pPFR cases have CBF changes that strongly track the changes in the pressure gradient (CPP), which is detected by a positive correction slope m>0 . Figure 2 depicts three such examples that exemplify pPFR: perfusion and CPP are positively correlated, as are perfusion and estimated CBF. Model estimated CBF (Figure 2, lower) correctly estimates the form of the perfusion trajectory from CPP. Trajectories may include transient excursions and variability, which are visible in the evolution of clinical indices (Mx, PRx) (right).

#### 3.2.2 Experiments identified with zero-PFR

**FIGURE 3:**
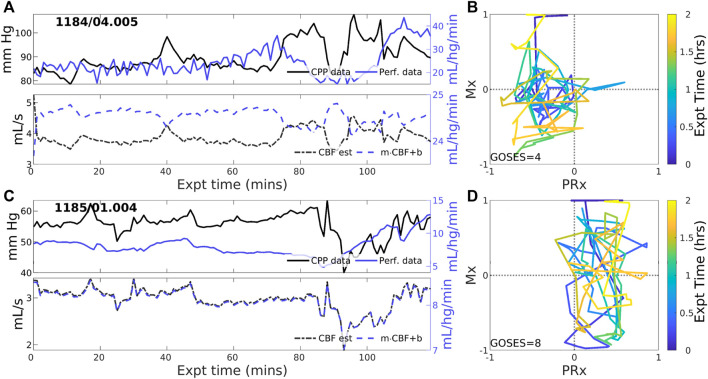
Two example experiments with zero PFR. The layout is the same as in Figure 2. Pressure (solid black) and perfusion (solid blue) data do not consistently coordinate over the 2-h experiment (upper panels of **(A,C)**). As a result, no suitable choice of Control parameters, corresponding to CA mechanisms, must alternate between positive and negative values to simulate these data for which no single PFR hypothesis suffices.

CBF-to-CPP coordination with an approximately zero-net mean is identified in 23 of 83 (27.7% of) experiments. These cases reflect statistical near-equilibrium between positive and negative PFRs, which may or may not result from decoupling of pressure and flow by active CA processes. Figure 3 illustrates several experiments where CPP (Figure 3, upper, black) has an irregularly structured relationship to perfusion data (blue) at both short and longer-term scales. CBF estimated from CPP cannot be corrected to consistently agree with perfusion data in these cases because the observed pressure-flow relationship is not stationary throughout the experiment. CPP and perfusion data (upper panels) evolve independently at times and may include periods of both positive and negative coordination. Highly variable Mx and PRx may potentially indicate reduced or range-inhibited CA function, discussed below in Section 3.3.

#### 3.2.3 Experiments identified with negative PFR

**FIGURE 4:**
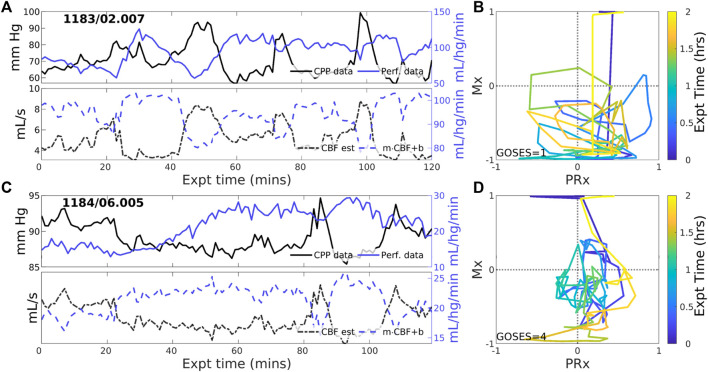
Example experiments with negatively-signed PFR. The layout is the same as the previous figure. Negative coordination between CPP (blue) and perfusion (magenta) observational data persists throughout many 2-h experiments. Model estimated CBF (blue, dashed) follows changes in CPP and *requires* a sign change in the posterior correction (dashed blue) match observed perfusion. Both Mx and PRx can be highly variable in these cases whose underlying causes are not known.

Negative CBF-to-CPP coordination dominates 22 of 83 (26.5% of) experiments. Two examples (Figure 4) illustrate persistently negative correlation between observed CPP (solid black lines) and perfusion (solid blue) at both short and longer-term scales. Such behavior violates pPFR-oriented model hypotheses so that estimated CBF (dashed black) requires correction with a *negative* slope (‘*flipped* and scaled’) to correctly represent perfusion. Several investigated EHR records (Supplementary Section S5) suggest reasons for nPFR appearance at hour timescales include metabolism and sedation, blood volumetric influences, and transitions across the limits of CA functionality.

### 3.3 ICHD data in relation to experiments

**FIGURE 5:**
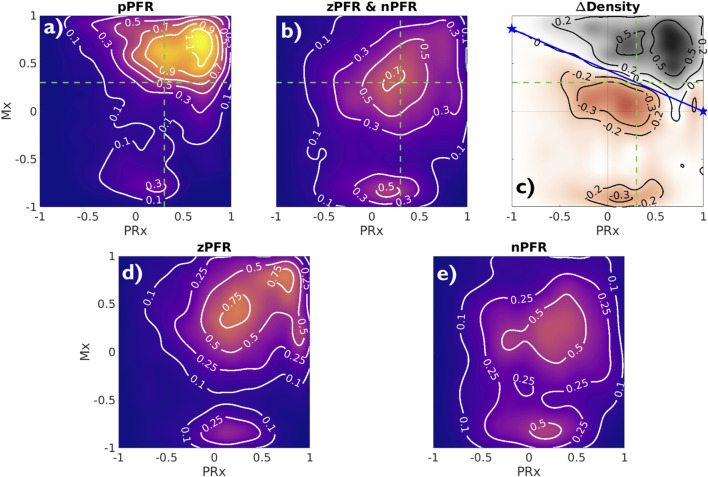
Joint Mx and PRx distributions and their difference Contours show distribution of (PRx, Mx) for pPFR (**(A)**, 27.3K points), zPFR **(D)**, 13.6K points), and nPFR **(E)**, 15.8K points) experiments, excluding unqualified data (PPA ≥ 5). For pPFR, the data density is highest at (PRx, Mx) = (0.7,0.75) where both pressure and flow aspects of autoregulation are impaired. Panel **(C)** shows the difference in densities of pPFR and non-pPFR **(C)** indices, identifying the region Mx=0.43⋅(1−PRx) dominated by pPFR cases. Green lines represent current index thresholds of 0.3 above which CA is assumed to be impaired; delineation based on PFR is a consistent joint consideration of those thresholds. The corresponding zPFR and nPFR distributions are distinct (bottom row). The zPFR data include both strongly positive and negative Mx values, whereas nPFR data consist of near-neutral positive Mx and strongly negative Mx.

Analyzing observational data through the lens of model-identified PFRs can better characterize ICHD and associated properties. Figure 5 empirically establishes the connection between PFRs and CA metrics, Mx and PRx. The aggregated joint (PRx, Mx) distributions for pPFR and non-pPFR experiments (left and center) differ significantly (two-sample K-S test (Wasserman, 2006), p<0.001 , D≈0.335 ). This difference (right) suggests a familiar, interpretable discrimination of PFRs using CA indexes. Particularly, pPFR experiments are delineated by joint (Mx, PRx) values (right, blue line), generalizing the independent thresholds on Mx and PRx for CA function (green lines). Note that parameters defining the linear discrimination depend on the window scheme used in Mx and PRx calculation.

High Mx values associated with pPFR correspond precisely to hypothesis of impaired CA, while remaining experiments explain the central mass of data around (PRx, Mx) = (0.3,0.3). The data associated with zPFR (bottom left) and nPFR (bottom right) experiments are also distinct, with the former being more variable and including both high and low values of Mx. This supports the hypothesis that longer-time pressure-flow decoupling arises from a balance of local positive and negative pressure-flow coordination which may be extreme in either direction. However, the nPFR subset (bottom right) lacks the presence of strongly positive Mx, so that their distribution includes a broad, neutral center near (PRx, Mx) = (0.27,0.2) and a more extreme negative center near (0.152,−0.82) . Within nPFR-identified data, the relative proportion occupied by the Mx-negative center decreases greatly when using longer averaging windows, which suggests the involvement of processes acting at sub-minute scales such as dynamic aspects of CA (Claassen et al., 2021).

Identifying PFRs as above depends on CBF/perfusion data and it is desirable to seek characterization of PFRs in more common data. For example, short-timescale autoregulatory responses to extra-hemodynamic influences (Briggs et al., 2022) might be detectable when CA is functional within non-pPFR cases. However, joint measurement data are broadly distributed at the 2-h timescale, and such variability limits discriminatory data analysis. Characteristics of PFRs are instead pursued among observed states they associate with. Perfusion is excluded from the following analysis due to its rarity and dependence of its values on patient- and placement-specific properties.

**FIGURE 6:**
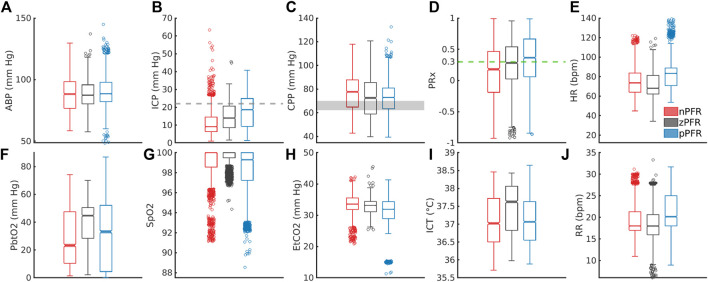
Distribution of 1-min averaged data in PFR-identified experiments. In the top row, panels **(A–E)** show ABP, ICP, CPP, PRx, and heart rate (HR), respectively. In the bottom row, panels **(F–J)** show P_bt_O_2_, SpO_2_, EtCO_2_, ICT, and respiratory rate (RR), respectively. **(C)** CPP medians exceed the protocol target range of 60–70 mm  Hg (grey). Both brain tissue oxygenation **(F)** and ICT **(I)** are elevated in zPFR relative to other data, although this may also reflect differences in patient care during intervals selected for experiment. Fully characterizing nPFR likely requires finer temporal and patient-specific analysis to identify the respective influences of high and low ICP, EtCO_2_, both systemic and brain-tissue metabolic factors.

PFR classification over 2-h windows summarizes significant short-term variability at 1-min timescales. Figure 6 shows the corresponding distributions of ICHD (Figures 6A–E) and non-hemodynamic variables (Figures 6F–J). All distributions except ABP are distinct (pairwise two-sample KS tests, p<0.005 ) with significant differences in median (pairwise two-sample t-tests, p<0.05 ) except: nPFR and pPFR have similar ICT, and SpO_2_ is tightly centered near 100% in all three groups. Positive PFR data indicate: elevated ICP, RR, and HR; reduced CPP; instances of reduced tissue oxygenation; and a majority of PRx values indicating impaired CA. The zPFR data link to moderate ICP and diminished CPP with moderate positive PRx centered near 0.3, the current threshold of CA function. The zPFR features the highest median tissue oxygenation under similar CPP to pPFR cases. Apart from the curiously elevated ICT among zPFR data, these characterizations conform with consequences of functional and impaired CA, respectively.

The nPFR data, in contrast to zPFR and nPFR, have lower PRx values that indicate functional CA and increased density at both low and super-critical ICP: over 15% of nPFR ICP is hypertensive ( >20 mm  Hg) while the median is ∼ 9 mm  Hg. This multimodal structure suggests that nPFR comprises multiple sub-dynamics involving flow-related effects not quantified by PRx. Compared with other cases, nPFR cases show reduced median tissue oxygenation paired with elevated CPP and the highest incidence of SpO_2_ < 92%. The data suggest nPFR characterization includes CA-mitigated flow to limit hyperemia (at low ICP/high CPP) and hypoxia (with low P_bt_O_2_ in response to further reduced SpO_2_). However, capnometry (EtCO_2_, *in lieu* of paCO_2_ (Razi et al., 2012)) is coherent across PFRs, with medians differing by ∼ 2 mm  Hg and extreme values linked to outlier measures of pulse oximetry and cardiopulmonary rates. Overall, the 1-min analysis considered here does not exclude volume-driven intracranial hypertension as a possibility in high ICP cases, as this requires finer temporal resolution to pinpoint causal influence.

### 3.4 Results summary and conclusions

Hypothesis-oriented experiments and PFR-differentiated data analysis found that:

1. Dynamics involving pressure-driven flow, pPFR, appeared throughout 46% of the experiment intervals (41 of 83) that were consistent with model assumptions. Remaining experiments divide about equally between nPFR (27%) and zPFR (28%) categories and include dynamics in violation of basic fluid mechanical rules governing pressure-driven flow. Hour-scale dynamics of zPFR cases typically feature an equilibrium between pPFR and nPFR modes with both high and low Mx values. Here, CPP-CPP coordination varies between positive (high Mx) and negative (low Mx) phases rather than comprising locally decoupled signals (Mx ≈ 0).
2. Computational ICHD models fitting the pPFR domain extended to the nPFR domain with minor modification because their physical assumptions are mathematically equivalent up to a sign change. A similar approach may be pursued for zPFR cases composed from mixed pPFR/nPFR dynamics by changing assumptions (and model signs) coincident with changes in PFR.
3. Pressure-flow identities could be inferred from the data through clinical indices Mx and PRx. Most nPFR experiment data has PRx values below 0.15, while the majorities for pPFR and zPFR experiments were above 0.25. Highly variable dynamics of both zPFR and nPFR cases are captured in trajectories of Mx and PRx. Joint use of PRx and Mx demarcates pPFR and introduces a novel method to identify impaired CA that generalizes current, independent index thresholds that may be insufficient (Figure 5, right).
4. The nPFR cases associate with both intracranial hypertension and low ICP, and nPFR-related dynamics may persist for multiple hours. About 15% of nPFR data feature ICP > 20 mm  Hg while half have ICP less than 10 mm  Hg under similar CPP. Compared with pPFR and zPFR, data of nPFR have reduced tissue oxygenation and highest incidence of SpO_2_ < 92%, which may be explained by CA-mitigated flow to limit hyperemia and hypoxia. These findings evince multiple nPFR sub-dynamics that plausibly discern between metabolically-driven flow at low ICP and volume-driven behavior at elevated ICP.
5. Detailed examination of patient trajectories (Supplementary Section S5) suggests that state-dependent engagement of CA in addition to the metabolic environment (Figure 6) affect the dominant PFR, and possible volumetric influences could not be ruled out. Sedation, mannitol, and patient stimulation events strongly influence the ICHD state and remain confounding factors in simulation-based experiments.

## 4 Discussion

This work analyzed a novel neurocritical dataset containing cerebral blood flow (CBF) and cerebral perfusion pressure (CPP) to investigate hour-scale pressure-flow relationships (PFRs). These relationships are essential, underlying components of computational and conceptual models of hemodynamics, and required rare, previously unavailable continuous perfusion or blood flow data to validate over longer scales. The positive pressure-flow relationship (pPFR), typically assumed in hemodynamics models and the mean flow index (Mx), dominated 46% of the 83 2-h experiments explored across 11 patients. About 28% of experiments showed no or weak orientation in PFR (zPFR) as expected when cerebral autoregulation (CA) is functional and engaged; such dynamics were not expected to be accurately deterministically simulated. However, 27% of experiments were best described by a negative PFR (nPFR) not fitting physiologically justified dynamics. This behavior is of interest for pressure-guided TBI management because the common pPFR assertion fails in these cases.

The pressure-driven flow regime pPFR (Eq. 1) is tied to dynamics with dysfunctional, impaired, or un-engaged CA mechanisms where blood flow changes are driven primarily by the arteriovenous pressure gradient, CPP. The dynamics of zPFR (Eq. 2) include active decoupling of pressure and flow by cerebral autoregulation, so that neutral values of PRx (ABP to ICP correlation) and Mx (CPP to flow correlation) were anticipated. The nPFR cases featured neutral PRx and strongly negative Mx, while associated observational data (Figure 6) suggests multiple possible origins including flow driven by metabolic demand and volume-driven pressure dynamics. The associated differences in joint (PRx, Mx) distribution (Figure 5) also show that the PFR perspective aligns with and generalizes current thresholds for CA impairment. These developments aim to support improvement of mechanistic process models and refine clinical guidance from CBF observation.

### 4.1 Key findings

Assumptions of pressure-driven flow do not apply to negative PFR cases, which are characterized by *strongly anti-correlated CPP-CBF dynamics*. These dynamics are governed by distinct physiological processes and violate model assumptions in a precise, identifiable, and correctable way. Specifically, they reverse the orientation of predicted CBF amplitudes about the mean (see Figure 4), an error correctable by a strategic sign change in Eq. 4. Specifying the correct hypothesis for these cases demands a more comprehensive understanding of the nPFR dynamics. The three PFR regimes were associated with considerable differences among peripheral data (viz. P_bt_O_2_, ICT, and SpO_2_) and parameters (PRx, Mx), although distributions were broad at both intra- and cross-patient levels. *Investigation of available data with numerous sources of heterogeneity could not establish PFR classification criterion without direct knowledge of CBF.*

The linear segmentation of (PRx, Mx) parameter distributions provided PFR-discerning criterion Mx>0.43⋅(1−PRx) to qualify CA impairment under the hypotheses of pPFR. This delineation (Figure 5) separates dynamical regimes in a way that generalizes current thresholds (of 0.3) for CA impaired based on Mx and PRx, highlighting the benefit of combined over individual index use. Such thresholds are sensitive to index calculation window and should be considered in relation to timescales of interest. The analysis also illustrates that PRx and Mx convey different information about ICHD. For example, PRx better discriminates pPFR from other categories, while Mx best discriminates the nPFR data.

Strong correlations between CPP and CBF (high positive Mx) facilitate mechanistic modeling in PFR cases where CA function is absent and predictive modeling may be urgently needed for patient care. In contrast, statistically decoupled hemodynamical observations and targets (neutral PRx and Mx) preclude modeling of zPFR regimes, although these cases are less likely to benefit from hemodynamic forecasting because patient CA appears functional. Between those cases, nPFR presumably features multiple hemodynamic behaviors and cannot be *mechanistically* modeled within the pPFR framework. However, the anti-correlated CBF-CPP dynamics found in nPFR are simulatable with a simple change in the model and hypothesis.

**FIGURE 7:**
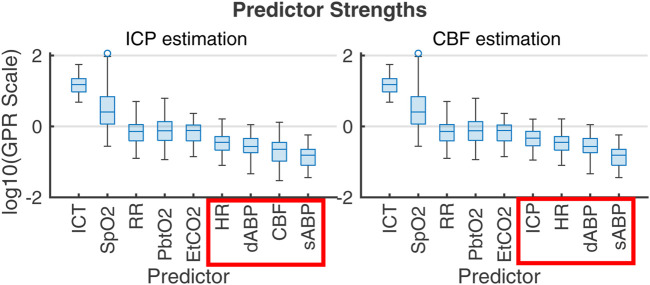
Gaussian process regression fitting of ICP (left) and CBF (right) from 1-s averaged data across the 83 2-h experiment intervals. Box plots give the distribution of scales of predictor influence on a logarithmic scale ordered decreasingly by median. ICHD model variables, indicated in red boxes, have low empirical predictive rank over the 2-h timescale. Importance rank is found by automatic relevance determination (ARD) (MacKay and Neal, 1994; Radford, 2012) using kernel function parameters associated with each predictor’s scale factor. The ranking is robust under alternate regression strategies (e.g., Lasso and Ridge Regression).

PFR-level differences in observational data could not be established independently of flow, highlighting the limitations of CPP as a CBF proxy and reinforcing the value of flow observation in the decision making knowledge environment. The plausible metabolic influences on CBF within nPFR motivates the use of empirical modeling to assess *which* clinically observable variables best relate to quantities of interest. Figure 7 ranks the influence of multi-modality monitoring data as predictors of ICP and CBF predictors in 83 experiment intervals as determined by Gaussian process regression (Williams and Rasmussen, 2006). For empirical ICP and CBF prediction, the most influential signals relate to metabolism/infection, respiration, and sedation rather than hemodynamics (outlined in red). Both nPFR and pPFR qualitatively share this ordering, which suggests their variables of interest might be approximated by a common model framework incorporating extra-hemodynamic factors over 1–2 h timescales.

### 4.2 Limitations and implications

Numerical simulations of this hypothesis-based analysis rely on specific data and data quality, potentially biasing the representation of patients with more abundant, complete, and clean data who may not reflect PFRs in the entire patient population. However, high intra-patient variability and long data series led to multiple PFRs appearing in most patients without a discernible pattern over time. The 166 patient-hours categorized from the 67 patient-days of data are insufficient for a robust analysis of PFR temporal evolution or injury-related differences, important topics for future study. The lack of population generalizability does not impair the study objective of validating model assumptions.

The scope of this work was limited to identifying PFRs rather than elucidating their causes. Specifically, it does not address the origins of these PFR dynamics which may involve specifics of other injuries, administered medications (sedatives and vasopressors), and other therapies (mechanical ventilation, fluid management). This limitation is attributable to data availability, as patient EHRs are needed to investigate and analyze the effects of these factors. The employed hemodynamic model did not include volumetric components or constraints. The exclusion of CSF, arguably the most significant dynamic volume component ignored, was justified by two key factors. Namely, its inclusion produces an unidentifiable model without additional data (e.g., CSF production and absorption rates), while the impacts of CSF dynamics on ICHD (Bothwell et al., 2019) are likely smaller and slower than those of CBF considered.

The presented PFR identification depended on *ad hoc* linear correction to equate global CBF estimates with local tissue perfusion data. This adjustment addressed measurement uncertainties and patient data variability while preserving signed coordination between pressure and flow. However, the true CBF-perfusion relationship is more complex and likely nonlinear as it depends on interacting factors including vascular stiffness, Circle of Willis geometry, and blood flow distribution at the probe site. A dedicated study comparing localized thermodiffusively-measured perfusion to global CBF estimates (via transcranial Doppler measurement) at the middle cerebral artery remains important, particularly to rectify the influence of injury distribution and probe location on observed flow. Although perfusion data were screened for quality based on probe diagnostics, unidentified factors such as spurious trends caused by ABP, ICP, or CBF sensor drift cannot be completely dismissed. Minimal impact on the primary findings of this study is expected as it is improbable that such artifacts occurred consistently over hour-long timescales of multiple patients.

The pPFR, defined by known physiological mechanics, served as a natural null hypothesis for PFR classification along with zPFR, whose hemodynamics include pressure-independent CBF. However, half of zPFR-identified experiments showed highly variable alternation between nPFR and pPFR extremes (Figures 3C, D) rather than a more stable balance (Figures 3A, B) postulated by theory. Subintervals of some highly variable zPFR cases may be categorized as nPFR or pPFR, making them potentially modelable (discussed below) but partially unexplained. Of notable interest are the variability and dynamics in (PRx,Mx) space (Figures 2–4C, D). These as well as encounter-scale trajectories of PFRs may contain substantial diagnostic and physiological information. Factors relating the temporal co-evolution of CA functionality and ICHD states were not explored here.

This analysis identified that nPFR comprises dynamics that diametrically opposite the pPFR assumptions used in models and underlie Mx as a clinical measure of CA function. The nFPR regime served as an alternate hypothesis to the mechanistically formulated dynamics, leaving the processes behind nPFR dynamics unexplained. However, data analysis and chart reviews (Section 3.3 and Supplementary Section S5) strongly suggest the existence of multiple subdynamics linked to metabolic responses and hyperemia in addition to CA functional range and the effects of intervention and stimulation.

### 4.3 Modeling implications

ABP-only estimation of ICP (Stroh et al., 2021b) generated consistently erroneous trends and phase of ICP in some experiments. Error diagnosis could not be explained without perfusion data and motivated this work. Although the errors were correctable by negating the pressure-flow relationship (a sign change in Eq. 4), such modification contradicted physical assumptions of the model. Change of model hypothesis are now justified by examining perfusion data: a sign change in Eq. 4 is necessary for dynamics governed by nPFR rather than pPFR. The change specifically addresses nPFR cases where dynamics are opposite to the pressure-driven flow assumed by the model. Physiologically, the correction corresponds to CA modulating flow opposite to CPP changes. This altered hypothesis pragmatically extends the pressure-driven flow model framework to nPFR dynamics. Despite the computational convenience of this approach, its pressure-governed formulation mis-represents, e.g., the volume-driven pressure and metabolically-driven flow regimes suggested by the data (Section 3.3 and Supplementary Section S5).

Harmonizing nPFR and pPFR assumptions into a common framework requires a method for correctly choosing which hypothesis applies for a particular patient, state, and time. However, a mechanistic representation is complicated by the distinct nPFR sub-dynamics and extra-hemodynamic CA stimuli that remain incompletely characterized. An accessible alternative is to wrap the current implementation in a machine learning (ML) layer (Farchi et al., 2021; Levine and Stuart, 2021) which predicts the PFR and necessary sign changes from training data and other relevant streams of patient observation. This approach leverages existing mechanistic ICHD models and their modification to extend model applicability to clinically-relevant, real-world cases.

### 4.4 Practical implications

CPP/ICP-oriented therapies rely on assumptions valid under pPFR that may not be valid for nPFR patient dynamics. Within zPFR dynamics where functional CA modulates flow, manipulation of CPP is only likely to directly influence CBF if CA response is altered, by e.g., by moving pressures across the limits of CA function. In nPFR dynamics, however, use of CPP to proxy CBF (Aries et al., 2012; Eriksson et al., 2012) incorrectly assumes positive pressure-flow coordination. ICP/CPP-based therapies may not ensure adequate perfusion in nPFR dynamic regimes which include flow/volume-driven pressure and metabolically-driven flow. In the former, interventions that manage intracranial volume budget (CSF drainage, heartrate) could be effective strategies for optimizing perfusion rather than raising ABP, particularly if the blood-brain barrier is compromised (Nordström, 2005). In the latter, which are postulated to feature low ICP (Figure 6B), CPP manipulation may unnecessarily disrupt endogenous flow regulation.

The ICHD system variables (CPP, CBF and perfusion, and the set of CA functions) warrant careful treatment of causal relationships, which can be overlooked when analyzing states rather than trajectories. Mx and PRx are CPP-CBF and ABP-ICP correlations, respectively, which measure different functional aspects of CA without causal direction between variables. Considering patient trajectory through the PFR perspective offers a more granular, dynamical perspective and incorporates causal logic into ICHD relations in contrast to separate use of Mx and PRx. Unlike pressures that influence the intracranial volume budget, blood flow contributes to it directly. Observations of CBF changes in the context of the pressure environment may also improve metrics and targets for TBI management that account for volume (viz. the Lund concept (Grände, 2006) and implicitly CPP_opt_ (Donnelly et al., 2018)). More long-time observation of CBF is required to investigate whether CPP/ICP-oriented manipulations result in appropriate perfusion under nPFR dynamics when CPP fails to proxy blood flow. The PFR perspective may also be valuable for discriminating which patient ICHD behaviors benefit from vascular protection by ICP-targeting therapies vs flow-optimizing CPP-optimizing targets, as (Howells et al., 2005) found management outcomes depended on patient autoregulatory state. From a wider perspective that sees CBF itself as proxy for oxygenation, PFR classifcation may also find use in extending oxygenation-guided therapies e.g., (Gouvêa Bogossian et al., 2022).

### 4.5 Concluding remarks and future work

The baseline assumption of positive pressure-flow coordination affects neurocritical care of TBI patients because ICP- and CPP-targeting guidelines aim to ensure adequate brain tissue perfusion through pressure management. This work analyzed those underlying assumptions for consistency against continuous, invasively-monitored TBI patient data, employing a physiological model to classify pressure-flow relationships based on assumption appropriateness. It revealed ICHD categories explained by whether CA processes were functional and found additional dynamics with strongly negative pressure-flow coordination sustained over long timescales. A simple yet unanticipated modification of assumptions was required to model negative pressure-flow coordination that may involve strong metabolic or volumetric drivers. The necessity explains the inaccuracy of CBF-less ICP model predictions that motivated this study, but it implies a similar misconception may exist in TBI management through pressure targets alone. The results bring into question the interpretation of highly negative Mx values in CA assessment, as they relate to different physiological hypotheses than positive and neutral values. Conclusions also motivate the use of CBF observation for personalizing neurocritical care to CBF and other observations. As PFRs distinguish hemodynamics by incorporating pressure and flow, and therefore generalize Mx and PRx (Figure 5), they are means toward that development. The mathematical formulae in ICHD models can be trivially modified to account for nPFR dynamics without understanding the mechanisms underlying their origin. Identifying the underlying drivers, causes, and covariates of particular PFR dynamics were not part of this work. Such a task ultimately require analyzing neurocritical monitoring data of a larger cohort in the context of patient, care, and observation heterogeneities gathered from EHR data including injuries, ventilator settings, sedation, and CSF maniuplation. Subsequent investigations focused on identifying the causes and physiological mechanisms of nPFR will contribute to a more nuanced understanding of PFRs and improve reliability of predictive decision support tool for cases currently outside the model framework.

## Data Availability

The data analyzed in this study is subject to the following licenses/restrictions: Data used in this work comprise high frequency timeseries and waveforms gathered during prospective clinical trial (https://tracktbinet.ucsf.edu/). Data access may be formally requested through the TRACK-TBI program at https://tracktbi.ucsf.edu/collaboration-opportunities. Requests to access these datasets should be directed to https://tracktbi.ucsf.edu/collaboration-opportunities.
