# Supplementary material for: Intracranial pressure-flow relationships in traumatic brain injury patients expose gaps in the tenets of models and pressure-oriented management
Source: Front Physiol. 2024 Aug 12;15:1381127. doi: 10.3389/fphys.2024.1381127 (PMC11345185; doi:10.3389/fphys.2024.1381127)
Supplement: Supplementary file 1 [file Presentation1.pdf]

Supplemental Sections to: Intracranial pressure-flow relationships in traumatic brain injury patients expose gaps in the tenets of models and pressure-oriented management

JN Stroh[1,2*], Brandon Foreman[3,4], Tellen D Bennett[1,5], Jennifer K Briggs[2], Soojin Park[6,7], David J Albers[1,2,6]

1 Department of Biomedical Informatics, University of Colorado Anschutz Medical Campus, Aurora, CO, USA
2 Department of Bioengineering, University of Colorado Denver |Anschutz Medical Campus, Denver, CO, USA
3 Department of Neurology and Rehabilitation Medicine, University of Cincinnati, Cincinnati, OH, USA
4 Gardner Neuroscience Institute, University of Cincinnati, Cincinnati, OH, USA
5 Pediatric Intensive Care, Children's Hospital of Colorado, Aurora, CO, USA
6 Department of Biomedical Informatics, Columbia University, New York, NY, USA
7 Department of Neurology, New York Presbyterian/Columbia University Irving Medical Center, New York, NY, USA

* jn.stroh@cuanschutz.edu

Supplemental Section S1

Assessing the internal consistency and effectiveness of CBF estimation requires numerical experimentation. However, the mechanistic relationship of CBF to the applied pressure gradient (∇p, the continuous-time equivalent of CPP) involves the unknown effects of CA. Simultaneous estimation CA and CBF is possible via inverse modeling centered on observed ABP, (P^{obs}). Optimization sequentially identifies CA parameters (α) within 1-minute windows until the model ICP estimate (I) agrees with observed ICP (I^{obs}) as measured by mean squared error (Eqns(1) below). The calculated CBF estimate ($\widehat{Q}$) is the optimal CBF ($\widehat{Q}^*$) in the model framework in that it reflects flow under a well-approximated pressure gradient via strategically optimized CA parameters ($\widehat{\alpha}^*$). Although implemented in discrete time, the continuous-time optimization can be expressed as $\widehat{Q}^* = \mathbf{F}(P^{\text{obs}} - I(\alpha^*), \alpha^*)$ for $\mathbf{F}$ (of Eqn.4 in main text) where

$$\begin{cases} I(\alpha) & = \mathcal{M}(P^{\text{obs}}; \alpha) \\ \alpha^* & = \text{argmin}_{\alpha \in \mathbb{R}^2_+} \|\text{mean}(I^{\text{obs}}) - \text{mean}(I(\alpha))\|^2. \end{cases} \tag{1}$$

Experiment definition here requires continuous input of ABP data for forward-time propagation, ICP data for model inversion, and CBF data for posterior correction and error diagnosis. The forward model $\mathcal{M}$ maps pressures to flow estimates on the basis of assumed parameters α.

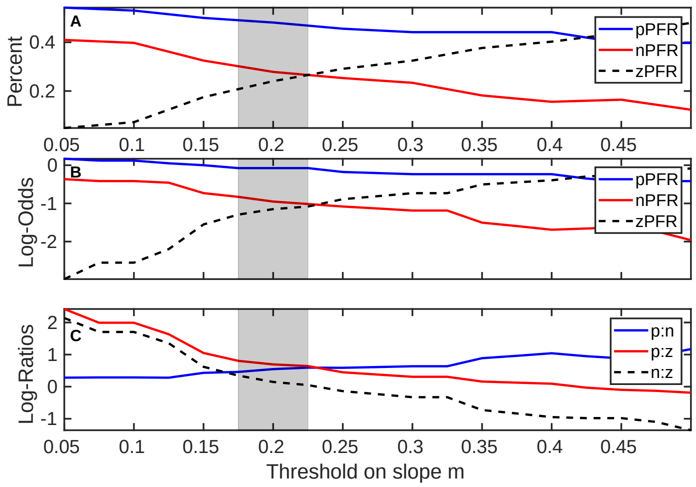

Figure 1: The threshold $|m| < 0.2$ used to discriminate PFR categories is influential on experimental results. Setting the threshold value too low eliminates the presence zPFR as a boundary between nPFR and zPFR. Experiments lacking estimated-to-observed flow are consequently corrected by ignoring dynamics and dubiously assigned nPFR or pPFR identities. Setting m too large, however, makes the zPFR category dominant at the expense of sensitivity to nPFR and pPFR identities. However, the choice of m cannot be determined from sensitivity/specificity trade-off analysis as true PFR identities are not known a priori. Among coarse choices $0 < m < 0.5$, the selected values $m \approx 0.2$ (shaded grey regions) shows (A) a moderate proportion of zPFR, (B) a local minimum in changes of odds for each PFR, and (C) a balanced pairwise odds among PFRs. Values of 0.25 and 0.3 are also reasonable. These higher values broaden the zPFR identity leaving nPFR and zPFR to comprise only extreme behavior, so the lower value 0.2 defines 'neutral slopes' more conservatively.

Supplemental Section S2

Table 1: Folder level summary of ICHD content of data in University of Cincinnati cohort of patients with recorded invasive regional perfusion (ml/hg/min), ABP (mm Hg), and ICP (mm Hg). Time t reports record interval in hours. PRx is calculated bedside on Moberg CNS monitor platforms using 30 samples of 10-second averages of ICP and CPP. Mx is calculated as part of this analysis using 30 samples of 12-second averages of perfusion and CPP. One epoch with less than one hour of valid perfusion data is omitted.

k	Data	GOSE	t	ABP	ICP	Perf	PRx	Mx
1	1073/01	1	0.0–9.5	80.2 ± 15.1	27.0 ± 6.0	11.9 ± 5.8	0.49 ± 0.4	0.62 ± 0.4
2	1073/02	1	9.8–18.5	85.9 ± 10.8	28.5 ± 5.8	46.0 ± 25.3	0.47 ± 0.4	0.40 ± 0.6
3	1073/03	1	18.6–34.0	86.6 ± 11.7	22.4 ± 7.2	85.3 ± 11.4	0.43 ± 0.4	0.01 ± 0.4
4	1073/04	1	34.1–38.0	97.1 ± 13.5	15.9 ± 8.4	149.2 ± 4.0	-0.16 ± 0.3	0.40 ± 0.1
5	1074/01	1	0.0–22.1	98.3 ± 26.0	19.0 ± 3.9	33.8 ± 41.5	0.12 ± 0.4	0.43 ± 0.4
6	1074/02	1	22.2–45.5	106.2 ± 11.9	23.9 ± 3.5	33.4 ± 22.0	0.12 ± 0.3	0.58 ± 0.3
7	1075/02	1	8.8–18.3	76.1 ± 10.1	17.5 ± 3.8	96.8 ± 25.0	-0.03 ± 0.3	0.03 ± 0.2
8	1075/05	1	57.1–99.0	93.3 ± 13.6	15.8 ± 4.8	1.7 ± 5.2	-0.04 ± 0.3	-0.02 ± 0.3
9	1086/01	1	0.0–25.3	83.3 ± 10.0	26.3 ± 6.2	29.9 ± 12.5	0.31 ± 0.3	0.64 ± 0.3
10	1086/02	1	25.4–28.1	91.3 ± 12.7	40.8 ± 6.2	40.4 ± 7.8	0.48 ± 0.4	0.45 ± 0.4
11	1086/03	1	28.4–32.4	96.7 ± 10.4	35.3 ± 8.8	45.8 ± 15.2	0.40 ± 0.3	0.71 ± 0.3
12	1086/04	1	32.5–47.4	93.3 ± 9.7	26.6 ± 8.9	33.9 ± 20.5	0.14 ± 0.3	0.75 ± 0.3
13	1086/05	1	47.4–71.2	92.4 ± 10.5	26.8 ± 6.3	37.7 ± 14.5	0.39 ± 0.4	0.55 ± 0.4
14	1086/06	1	71.3–96.2	88.4 ± 11.4	28.5 ± 4.2	30.9 ± 13.1	0.60 ± 0.3	0.58 ± 0.3
15	1086/07	1	96.4–100.0	104.7 ± 10.6	35.8 ± 3.3	35.5 ± 16.3	0.46 ± 0.3	0.53 ± 0.3
16	1086/08	1	100.7–107.7	62.8 ± 52.1	38.3 ± 5.0	17.8 ± 6.7	0.42 ± 0.4	0.09 ± 0.5
17	1140/01	5	0.0–94.8	79.1 ± 14.6	13.7 ± 4.9	14.5 ± 10.4	0.02 ± 0.4	0.25 ± 0.4
18	1141/01	1	0.0–105.4	79.1 ± 14.8	18.0 ± 4.5	13.0 ± 9.4	0.46 ± 0.4	0.29 ± 0.4
19	1160/01	1	0.0–24.1	83.5 ± 17.0	11.3 ± 2.9	22.0 ± 12.4	0.38 ± 0.3	0.45 ± 0.3
20	1165/01	1	0.0–17.1	88.8 ± 14.8	23.1 ± 6.2	22.0 ± 6.6	0.48 ± 0.3	0.36 ± 0.4

21	1166/01	3	0.0–25.7	84.4 ± 12.8	6.7 ± 4.2	25.7 ± 10.9	0.22 ± 0.3	0.46 ± 0.3
22	1166/02	3	25.7–46.5	94.0 ± 11.2	8.4 ± 4.7	23.4 ± 8.1	0.34 ± 0.3	0.46 ± 0.3
23	1166/03	3	46.5–70.2	84.6 ± 9.2	8.7 ± 4.4	21.7 ± 8.0	0.27 ± 0.3	0.54 ± 0.3
24	1166/04	3	70.2–91.3	104.4 ± 14.5	19.1 ± 7.2	14.8 ± 6.1	-0.12 ± 0.3	0.18 ± 0.4
25	1171/02	1	0.7–86.1	82.2 ± 13.6	8.2 ± 5.9	42.9 ± 2.9	0.13 ± 0.5	0.95 ± 0.2
26	1178/01	3	0.0–52.8	84.6 ± 12.1	14.1 ± 3.4	47.0 ± 14.5	0.14 ± 0.3	0.12 ± 0.5
27	1183/02	1	25.2–94.3	86.2 ± 14.7	13.5 ± 7.4	80.8 ± 36.2	0.08 ± 0.4	-0.58 ± 0.4
28	1183/03	1	106.3–133.5	96.4 ± 11.5	15.2 ± 8.8	62.8 ± 25.6	-0.00 ± 0.3	-0.36 ± 0.4
29	1183/04	1	133.5–136.9	78.9 ± 40.9	14.8 ± 6.0	69.6 ± 12.1	-0.18 ± 0.3	-0.07 ± 0.5
30	1184/01	4	0.0–32.5	82.2 ± 18.4	3.5 ± 2.1	40.4 ± 12.8	0.23 ± 0.3	0.16 ± 0.4
31	1184/02	4	32.5–60.0	79.3 ± 11.3	5.5 ± 1.9	20.1 ± 11.8	0.47 ± 0.4	0.42 ± 0.3
32	1184/03	4	60.0–86.7	75.7 ± 12.2	5.3 ± 2.0	26.5 ± 12.2	0.38 ± 0.4	0.31 ± 0.3
33	1184/04	4	86.7–110.8	88.5 ± 18.2	7.5 ± 2.5	28.6 ± 15.4	-0.13 ± 0.4	0.08 ± 0.4
34	1184/05	4	110.8–133.6	97.9 ± 10.7	6.7 ± 3.0	26.4 ± 15.1	0.02 ± 0.4	0.05 ± 0.4
35	1184/06	4	133.6–181.0	97.8 ± 17.6	7.1 ± 2.8	52.2 ± 40.5	-0.03 ± 0.4	-0.01 ± 0.4
36	1185/01	8	0.0–18.5	70.5 ± 12.9	10.7 ± 2.4	18.8 ± 22.4	0.17 ± 0.3	0.15 ± 0.3
37	1185/02	8	18.6–29.7	71.8 ± 9.5	14.4 ± 2.9	18.0 ± 4.3	0.14 ± 0.3	0.04 ± 0.4

Supplemental Section S3

Table 2: Simulation experiment results, including the signed scale coefficient (m) used to adjust model solutions for evaluation against data. RMSD values measure the difference between observed perfusion and estimated CBF adjusted by a linear correction with slope m (reported here in dimensional form that includes the magnitude of ratio between perfusion and CBF variability). Dagger-flagged PPA in rows indicate data of questionable quality excluded from aggregate analysis.

k	Expt	PPA	t_0	m	$\overline{\text{PRx}}$	$\overline{\text{Mx}}$	PFR
1	1073/01.08	0.0	7.5	+0.48	0.64	0.71	p
2	1074/01.07	0.0	9.1	+23.30	0.21	0.60	p
3	1074/01.09	$9.6^\dagger$	13.5	+22.84	0.03	0.71	p
4	1074/01.11	$7.9^\dagger$	16.6	+36.95	0.43	0.58	p
5	1074/01.12	$6.7^\dagger$	18.7	+14.48	-0.23	0.89	p
6	1074/02.03	$6.2^\dagger$	2.4	+12.49	0.23	0.48	p
7	1074/02.04	3.9	4.6	+3.11	0.09	0.73	p
8	1074/02.09	$4.8^\dagger$	13.2	+9.59	0.07	0.78	p
9	1074/02.10	2.4	15.3	+5.45	0.19	0.68	p
10	1074/02.12	2.8	18.9	+1.33	0.22	0.58	p
11	1086/01.07	0.0	16.1	+0.27	0.35	0.86	z
12	1086/01.09	0.6	20.3	-0.01	0.23	0.60	z
13	1086/01.10	0.3	22.5	+1.06	0.33	0.59	p
14	1086/05.04	1.1	4.6	+1.39	0.56	0.72	p
15	1086/05.11	0.0	19.6	-0.04	0.66	0.74	z
16	1086/06.02	0.0	2.1	+10.59	0.73	0.59	p
17	1086/06.03	0.0	4.2	-0.43	0.49	0.43	z
18	1086/06.04	0.0	6.4	+2.04	0.59	0.84	p
19	1086/06.05	0.0	8.5	+2.00	0.76	0.57	p
20	1086/06.06	0.0	10.6	+0.81	0.61	0.63	p
21	1086/06.07	0.0	12.8	-0.10	0.75	0.73	z
22	1086/06.09	0.0	17.0	+3.40	0.60	0.85	p
23	1086/06.11	0.0	21.3	+3.72	0.50	0.58	p
24	1140/01.08	0.0	11.8	+0.15	0.25	0.44	z
25	1141/01.19	0.0	18.8	-0.12	0.74	0.29	z
26	1141/01.72	0.0	68.3	+0.05	0.82	0.26	z
27	1141/01.73	0.0	70.5	-0.52	0.55	0.22	**n**
28	1141/01.75	0.0	74.2	-0.11	0.65	-0.02	**n**
29	1141/01.76	0.0	76.4	+0.67	0.64	0.30	p
30	1141/01.78	0.0	80.8	+5.29	0.40	0.31	p
31	1141/01.84	0.0	87.3	+0.60	0.51	0.53	p
32	1141/01.93	0.0	97.9	+0.10	0.20	0.25	z
33	1160/01.03	0.0	7.3	-2.44	0.38	0.61	**n**
34	1165/01.06	1.3	7.4	+0.93	0.62	0.51	p
35	1166/01.12	0.0	20.0	+1.52	0.16	0.60	p
36	1166/01.13	0.4	22.2	+1.29	0.18	0.68	p
37	1166/02.02	2.2	0.9	-0.09	0.30	0.49	z
38	1166/02.03	3.4	3.0	-0.02	0.41	0.51	z
39	1166/02.06	3.2	9.5	+6.45	0.42	0.63	p
40	1166/02.08	1.3	12.2	-0.31	0.20	0.59	z
41	1166/03.03	0.0	2.5	+0.08	0.20	0.51	z

42	1166/03.04	0.0	4.6	-0.81	0.24	0.49	**n**
43	1166/03.08	0.0	10.8	+0.95	0.39	0.69	p
44	1166/03.10	0.0	15.1	+0.24	0.18	0.61	p
45	1166/03.12	0.0	19.4	+0.42	0.35	0.79	p
46	1166/03.13	0.0	21.5	+0.74	0.11	0.68	p
47	1166/04.02	0.0	2.1	-0.61	0.05	0.33	z
48	1166/04.04	0.0	6.7	-0.18	-0.08	0.11	z
49	1166/04.05	0.0	8.8	-0.65	-0.16	0.26	z
50	1166/04.06	0.0	11.0	-0.52	-0.22	0.16	**n**
51	1166/04.09	0.0	17.5	-0.23	-0.25	-0.07	**n**
52	1183/02.04	0.0	17.8	-0.90	0.19	-0.65	z
53	1183/02.07	3.0	22.9	-0.94	0.24	-0.53	**n**
54	1183/02.11	3.2	30.4	-3.87	0.07	-0.41	**n**
55	1183/02.12	2.0	32.5	-1.70	0.05	-0.65	**n**
56	1183/02.16	3.7	42.3	-3.69	0.01	-0.70	**n**
57	1183/02.19	1.2	49.1	-1.25	0.00	-0.31	**n**
58	1183/02.20	0.0	52.2	-5.86	0.12	-0.38	**n**
59	1183/02.24	0.6	60.1	-0.25	0.07	-0.65	z
60	1183/03.05	1.6	10.7	+2.00	0.10	-0.44	p
61	1183/03.06	3.1	13.0	+3.04	0.00	-0.16	p
62	1183/03.08	1.9	17.1	+12.39	-0.05	-0.31	p
63	1183/03.09	$4.1^{\dagger}$	20.5	+1.89	-0.08	-0.06	p
64	1184/01.09	4.0	9.2	-0.24	0.07	0.18	**n**
65	1184/01.11	0.0	13.4	-0.39	0.16	0.33	z
66	1184/01.12	0.0	15.5	+0.90	0.06	0.54	p
67	1184/02.02	0.7	0.7	-0.03	0.13	0.63	z
68	1184/02.16	0.8	20.1	+11.45	0.69	0.73	p
69	1184/02.19	1.3	25.2	-2.53	0.57	0.37	**n**
70	1184/03.03	2.4	4.2	+7.00	0.72	0.63	p
71	1184/03.04	3.6	6.4	+3.43	0.66	0.42	p
72	1184/03.06	$4.3^{\dagger}$	10.6	-6.90	0.40	0.53	**n**
73	1184/04.03	4.0	3.8	-2.73	-0.25	0.28	**n**
74	1184/04.05	0.0	8.1	-0.14	-0.37	0.16	z
75	1184/04.09	0.0	14.4	-0.37	-0.14	0.13	**n**
76	1184/04.11	0.0	17.7	-0.73	-0.04	-0.11	**n**
77	1184/05.09	0.0	12.6	-0.45	0.03	0.06	**n**
78	1184/06.01	0.0	0.0	-0.48	0.40	-0.09	**n**
79	1184/06.05	0.3	6.2	-13.68	0.13	-0.09	**n**
80	1185/01.02	0.0	1.0	+0.60	0.10	0.34	p
81	1185/01.04	0.0	3.7	+0.21	0.31	0.06	z
82	1185/01.05	1.2	5.9	+0.08	0.29	0.07	z
83	1185/01.06	0.0	8.0	-0.20	0.22	0.28	**n**

Supplemental Section S4

Table 3: Experiment result summary. Columns tabulate PPA limits, the number of patients represented, the number of represented epochs (folder of patient data), the number of nominal 2-hour experiments conducted, and the corresponding percentage of experiment identified with pPFR (p), zPFR (z), nPFR (n). Experiment results are not influenced by CBF quality measured by PPA values.

	N_{patients}	N_{epochs}	N_{expts}	% PFR (p/z/n)
All	11	23	83	46/28/27
PPA≤5	11	22	78	44/29/27
PPA≤2	11	21	61	39/33/28
PPA≤1	10	19	52	38/35/27

Supplemental Section S5

Patient #04-1166 data define several experiments over 4 epochs, two of which are shown in Fig2.

Successful modeling stops around hour 70.15 of their record (between entries 46 and 47) as the dominant regime changes from pPFR to zPFR and nPFR. During pPFR-labeled experiments, the highly-sedate patient suffers autonomic dysregulation characterized by irregular transient ICP elevation after 65 hours. Flow dynamics of this period track the pressure gradient (positive Mx) while perfusion is high (21.5 mL/hg/min) and CPP is ample (75.5 mm Hg), although pressures are low (mean diastolic ABP$\approx$55 mm Hg), subcritical ICP$\approx$8.5 mm Hg). ABP elevation during the second epoch (lower panel) results in the engagement of CA – thereby decreasing CBF – once the CPP exceeds the lower limit of CA (estimated at about 70 mm Hg.). Model-based experiments fails in these cases because the model lacks the hidden state-dependent (and likely individualized) limits at which autoregulatory engagement occurs or vanishes.

Patient #04-1183 is featured in an nPFR experiment (Figure3A,B; experiment table entry 53) characterized by a nearly exact negative pressure-flow relationship throughout the interval. The appearance of nPFR here may be explained by volumetric changes resulting from stimuli-instigated tachypnea. Namely, a decrease in CO_2 initiates vasoconstriction, directly decreasing CBF while indirectly increasing CPP (by lowering ICP) through reduction of vessel-occupied volume. Model estimation fails to account for cardiovascular rate changes and volumetric feedback between vasoregulation and ICP, although MMM data is insufficient at this specific time needed to confirm this (EtCO2 recording is absent at the relevant period).

Patient #04-1165/01 Experiment 006 is pPFR case (not illustrated) with a delayed relationship between ICHD factors at local scales but a positive coordination at long timescales between pressure and flow. Observed dynamics in this interval are primarily driven by therapeutics, beginning with a decreased of both sedation and vasopressors around 10 minutes, and promptly increased following the hypotensive event. Increased ABP and ICP yield a net increase in CPP, until about 38 minutes into the simulation when mannitol is given to regulate intracranial hypertension. The late, delayed increase in CBF following CPP decrease results from external management rather than endemic processes.

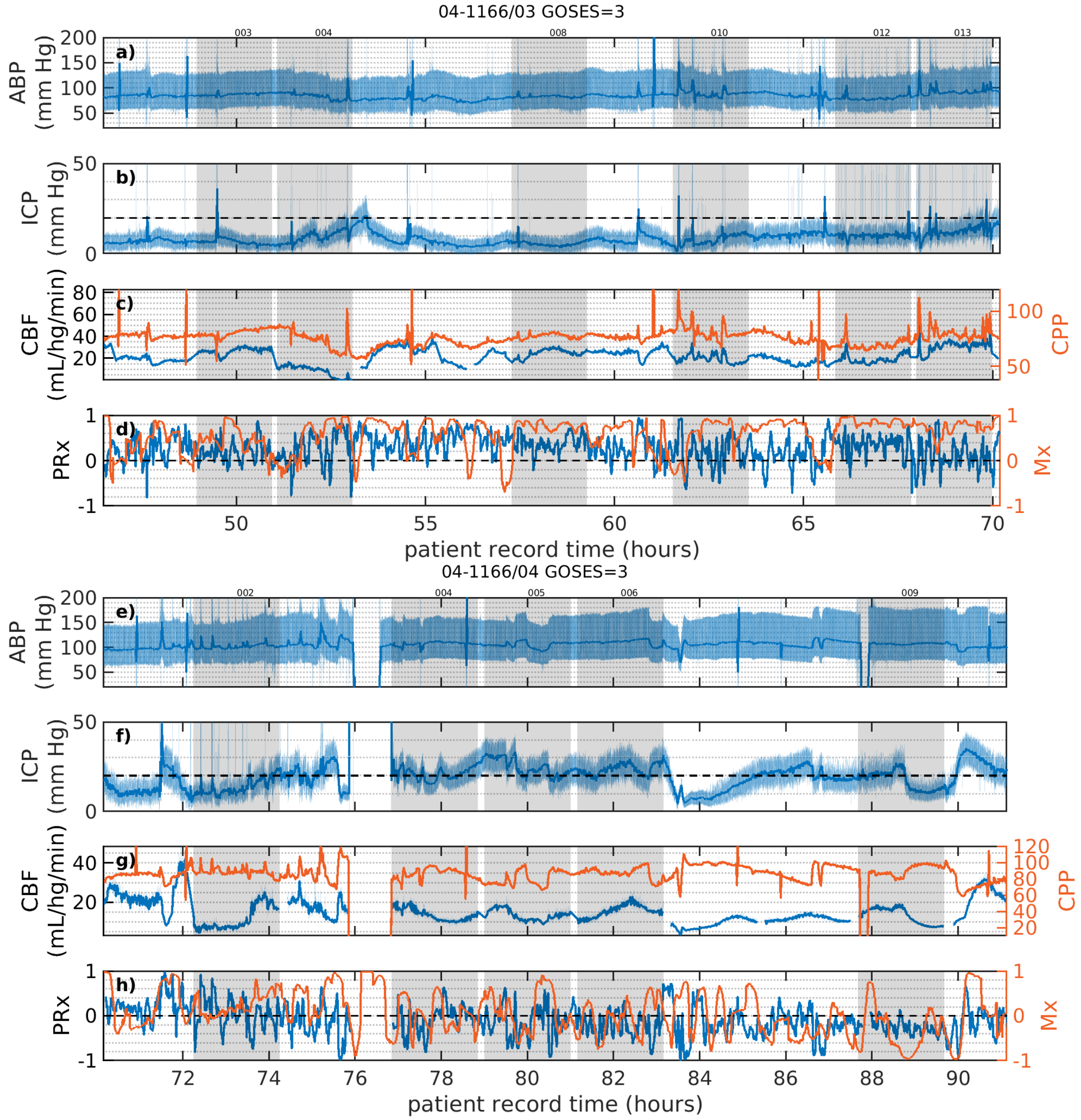

Figure 2: Two epochs of longitudinal data for patient 04-1166 contextualize PFR evolution and numerical experiments. Panels a–d and e–h present timeseries of from epochs 3 and 4 corresponding to summary table rows 24 and 25, respectively. Left axes and blue curves correspond to MMM data of patient 1166 during the ∼2-day period starting 46.5 hours into hospitalization. Dashed line in the second rows indicate 20 mm Hg ICP. Grey boxes throughout denote experiments: the last four experiments in the upper panel (rows43–46 of experiment tables) are pPFR-governed while all experiments in the lower panel indicate nPFR. Experiment 008 in panels a–d corresponds to the lower pPFR example of main text Figure 2C,D.
